# Diversity, equity and inclusion in One Health could crucially support functional health security by fostering prevention, but a change in mindset is needed

**DOI:** 10.1186/s42522-025-00175-3

**Published:** 2025-11-11

**Authors:** Claudia Robbiati, Antsa Miarisoa Andriamandroso, Heidi Auerswald, Mónica Berger González, Natalia Cediel Becerra, Maria Grazia Dente, Nguyen Thi Dien, Julie Garnier, Diana Onyango, Tamara Riley, Kim Laura Weiszhar, Andrea Sylvia Winkler, Robyn Alders

**Affiliations:** 1 Italian National Institute of Health (Istituto Superiore di Sanità), National Center for Global Health, Rome, Italy; 2 Independent Consultant in One Health Integration, Antananarivo, Madagascar; 3 Istituto Zooprofilattico Sperimentale dell’Abruzzo e del Molise (IZSAM), Teramo, Italy; 4 Universidad del Valle de Guatemala, Guatemala City, Guatemala; 5 Facultad de Ciencias Agropecuarias, Universidad de La Salle, Bogotá, Colombia; 6 Department of Sociology, Faculty of Social Sciences, Vietnam National University of Agriculture, Hanoi, Vietnam; 7 Odyssey Conservation Trust, Bakewell, Derbyshire, UK; 8 Farm Africa, Nairobi, Kenya; 9 Yardhura Walani, National Centre for Epidemiology and Population Health, The Australian National University, Canberra, Australia; 10 Department of Neurology, TUM University Hospital, and Center for Global Health, TUM School of Medicine and Health, Technical University of Munich (TUM), Munich, Germany; 11 Department of Community Medicine and Global Health, Institute of Health and Society, University of Oslo, Oslo, Norway; 12 Department of Global Health and Social Medicine, Harvard Medical School, Boston, MA USA; 13 Development Policy Centre, The Australian National University, Canberra, Australia

**Keywords:** One Health, Diversity, Equity, Inclusion, Health security

## Abstract

In recent years, One Health (OH) has taken the lead as a systems-oriented method to foster transdisciplinary, multisectoral, and multi-actor action to promote global health security. However, operationalizing the OH approach is difficult since it requires equitable collaboration, communication and information sharing. The One Health High-Level Expert Panel’s (OHHLEP) definition of OH lists key underlying principles at its core, which align with principles of diversity, equity and inclusion (DEI), and establishes that the application of the definition is incomplete without the adoption of these key principles. In this paper, we argue that, by overcoming the barriers that hamper OH adherence to DEI principles, the operationalization of the OH approach could be significantly enhanced to support global health security. We built on the key underlying principles included in the OHHLEP 2022 definition of OH to map barriers preventing its full-scale implementation and to identify inclusive avenues to promote the compliance of OH with its core principles. A scoping review of the literature and consultations with 10 OH professionals from different disciplinary backgrounds, regions of the world and levels of experience were performed. The barriers to the full adherence of OH to its underlying principles that emerged from this study were grouped into five categories: mindset, behaviors and awareness, conceptual, structural, power dynamics, and governance and implementation. Crucially, the engagement of diverse sectors and disciplines notably the environmental and social sciences; of different actors from communities and young people to donors and OH professionals, including the private sector; and of underrepresented groups, such as Indigenous peoples, farmers, fishers, representatives from low- and middle-income countries, and especially women across these groups, all intimately connected to the drivers of emerging health threats, is not only critical for realizing DEI principles in OH, but also to promote more effective prevention strategies and thus enhance global health security.

## Introduction

One Health (OH) represents an integrated and holistic approach (i.e., it is not a discipline in itself) that mobilizes multiple disciplines, sectors and stakeholders, which largely focuses on tackling infectious but also noninfectious diseases with an emphasis on the emergence of threats at the human–animal–environment interface [1]. In recent years, OH has taken the lead as a systems-oriented method to foster transdisciplinary (crossing disciplinary boundaries), multisectoral (involving different sectors), and multi-actor (engaging different groups of actors) actions to promote global health security [2–5], with past epidemics and pandemics acting as a driver of more extended collaborations [6–7]. The current global health security architecture is focused mainly on response actions [8], and, despite the current efforts, there remains a gap in investing for prevention of epidemics and a shift to OH prevention of global health threats is urgently needed [9–11]. An efficient OH operationalization could support upstream prevention by addressing social, cultural, economic, political and ecological determinants and anticipate the emergence and re-emergence of challenges at the human–animal–environment interface [12–14]. However, operationalizing the OH approach is difficult since it requires equitable collaboration, communication and information sharing, as highlighted by *The Lancet* Series on One Health and Global Health Security [15]. The One Health High-Level Expert Panel’s (OHHLEP) definition of OH identifies key underlying principles at its core that align with concepts of diversity, equity and inclusion (DEI) and establishes that the application of the definition is incomplete without the adoption of the key principles [1]. However, these principles are often overlooked during the design and implementation of OH approaches [16, 17].

In this paper, we argue that by overcoming the barriers that hamper OH from adhering to its key underlying principles, the operationalization of the OH approach could be significantly enhanced to support global health security.

### Methods

**Fig. 1:**
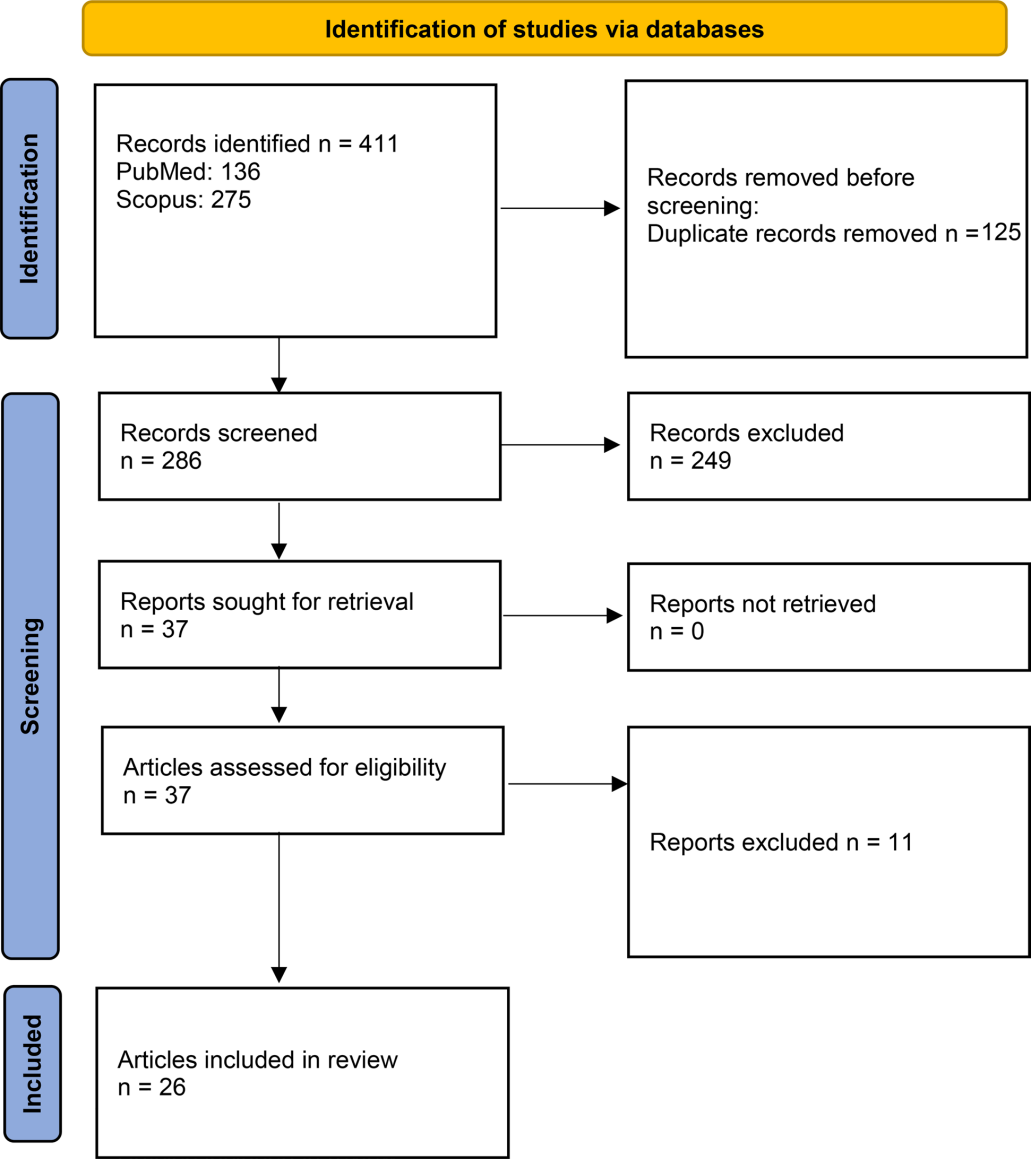
Flow chart of the scoping review

We built on the key underlying principles included in the OHHLEP definition of OH [1] (Table 1) to map barriers preventing its full-scale implementation according to each principle and to identify inclusive avenues to promote the adherence of OH to its key principles. A scoping review of the literature (Fig. 1) was performed and the databases searched were:

Scopus: Search string “One Health” AND gender OR equity (Title/abstract/keywords).

Pubmed: Search string (“One Health“[Title/Abstract]) AND ((equity[Title/Abstract]) OR (gender[Title/Abstract]))

Any type of document published between the 1st of January 2018 and the 20th of November 2023, in English, with full-text available was included.

10 consultations were performed between June and September 2023 with OH professionals (women) belonging to the Women for One Health Network (https://wfoh.org/) whose primary discipline was animal health (*n* = 3), social sciences (*n* = 3), human health (*n* = 2), and ecosystem health (*n* = 2). Their geographical affiliation was Africa (*n* = 3), Europe (*n* = 3), Central and South America (*n* = 2), and the Asia-Pacific region (*n* = 2). Their work experience was spanning between young professionals (*n* = 3), mid-level (*n* = 3) and senior level (*n* = 4). A thematic analysis was performed to identify barriers to OH to adhere to its key underlying principles [1], which align with diversity, equity and inclusion principles and how to overcome them. Oral consent was obtained during the consultations and written consent for publication of the results was obtained from each of the consulted experts. 

**Table 1:** OH key underlying principles embedded in the OHHLEP definition [16] and the corresponding sections and panels in the Article

Key underlying principles from the OHHLEP definition	Article’s sections
Equity between sectors and disciplines	The interface is flawed (Panel 1)
Sociopolitical and multicultural parity (the doctrine that all people are equal and deserve equal rights and opportunities) and inclusion and engagement of communities and marginalized voices	Who sits at the OH table? (Panel 2)
Socioecological equilibrium that seeks a harmonious balance between human–animal–environment interaction and acknowledging the importance of biodiversity, access to sufficient natural space and resources, and the intrinsic value of all living things within the ecosystem	We are disconnected (Panel 3)
Stewardship and the responsibility of humans to change behavior and adopt sustainable solutions that recognize the importance of animal welfare and the integrity of the whole ecosystem, thus securing the well-being of current and future generations	All life is of equal concern (Panel 4)
Transdisciplinarity and multisectoral collaboration, which includes all relevant disciplines, both modern and traditional forms of knowledge and a broad representative array of perspectives	Holistic traditional knowledge systems are disrupted (Panel 5)

### The interface is flawed

OH should promote equity between sectors and disciplines by acting at the human–animal–environment interface to enable effective transdisciplinary and multisectoral collaboration. However, the reductionist evolution of the OH approach, stemming from veterinary and human medicine, only recently conceptually incorporated the environmental sciences [18], which remain poorly integrated in practice [19]. On the other hand, social sciences are the “missing link” in the OH approach, since they are often totally neglected in the operationalization of the approach, despite their fundamental role in supporting global health security [20–23]. As such, the OH interface remains flawed, anchored in the medicalization of health, and hindered by inadequate collaboration among epistemologically different disciplines and by siloing between sectors and disciplines. Institutional stakeholders are not always aware of the need for and benefits of the OH approach for the prevention and control of health threats, and of the multiplicity of sectors, disciplines and actors required to achieve successful outcomes. This paradigm has resulted in an inadequate focus on the socioecological drivers of health threats and inequity in resource allocation between different sectors and disciplines [24]. Moreover, resources and attention are disproportionally directed toward antimicrobial resistance (AMR) and zoonoses [25], leaving other critical threats under addressed. Therefore, a paradigm shift is urgently needed to effectively support genuine transdisciplinary and multisectoral collaboration, which must be based also on equity of resources distribution among the different sectors and disciplines.

#### Panel 1: Avenues to support equity of disciplines and sectors in One Health

Transdisciplinary and multisectoral collaborations must be implemented from the local level to the institutional level, to achieve a functional OH operationalization. However, disciplines and sectors vary in their terminology, tools, methods, perspectives, and ways of synthesizing the results, despite overlaps and similarities. To address these discrepancies, efforts are needed both conceptually and operationally to align terminologies and methodologies, for example through joint capacity building activities, while sufficient resources are allocated to support research, programs, and policy-making that address the holistic determinants of health threats. In this context, interventions should be reviewed and developed through a OH lens to strengthen collaboration of disciplines and sectors, also by leveraging programs, procedures and mechanisms where multiple disciplines and sectors are already engaged [26]. As an example in Italy, the Ministry of Health includes both public health and animal health services and this framework allowed for a more efficient OH surveillance of arboviral diseases, such as West Nile Virus [27].

### Who sits at the One Health table?

OH interventions should ensure sociopolitical and multicultural parity (the doctrine that all people are equal and deserve equal rights and opportunities) and the inclusion and engagement of communities and marginalized voices. The stakeholders engaged in the governance of OH interventions are most commonly research and government institutions and international organizations [25, 28]. For OH interventions to be effective, all the relevant actors, including communities, especially at-risk groups, the private sector and donors, must sit at the table from the outset to codesign, co-implement and share the benefits of the intervention [29]. This would allow a better understanding of the holistic determinants of the challenges addressed and the resources available. Barriers to the meaningful inclusion of all the relevant stakeholders are manifold and could be related to a mindset that is less prone to inclusion, personal interests, difficulties in collaboration, a lack of obvious incentives, and a poor understanding of the OH approach, among others. Currently, parity in OH is not ensured also in terms of the geographical and gender-related representation of the stakeholders. Despite low- and middle-income countries (LMICs) being hotspots of emerging risks at the human–animal–environment interface [30], stakeholders from these regions often lose prominence in research collaborations over time and are replaced by high-income country institutions, perpetuating global health power imbalances [28]. Gender disparities are similar and significant. In patriarchal societies, rural women—key contributors to maintaining safe and clean environments, managing households and livestock, and supporting food security—are often marginalized. They face limited access to education, land ownership, and political decision-making, which restricts their agency and decision-making power and undervalues their critical knowledge for successful OH interventions [31, 32].

#### Panel 2: Avenues to support sociopolitical and multicultural parity in One Health

Sociopolitical and multicultural parity and the inclusion and engagement of communities and marginalized voices in OH could be difficult given the heterogeneity of the actors involved. For true parity, for each intervention, all the relevant stakeholders should be identified and engaged through their chosen representatives. This could support access to decision-making power and to bring everyone’s perspectives in the development of projects, programs and policies. A OH facilitator could help to bridge the gaps between institutional and noninstitutional actors and should be appointed to navigate the complexity involved in coordination and communication with such a heterogeneous group. The facilitator could be a OH professional with in-depth knowledge, skills and competences related to OH and a broader understanding of the task at hand. OH facilitators would need to be familiar with the specific context and accepted by all the stakeholders. Ideally, OH facilitators should belong to an institution while maintaining active links with communities and be able to bring their needs, rights and perspectives to the table. A review of the literature about OH coordination mechanisms for vector-borne diseases (VBDs) interventions highlighted that to support coordination and collaboration among the various stakeholders, synergistic interaction mechanisms were created, such as working groups and committees, and connection agents, OH experts with a strong link with institutions and communities, emerged as the main facilitators of coordination and communication among stakeholders. These OH coordination mechanisms allowed targeting of the multidimensional drivers of VBDs, supported transversal capacity building and an holistic monitoring evaluation framework, and improved effectiveness and sustainability of the interventions [33]. Policy-makers and donors should incorporate OH facilitators into project and program design and implementation, ensuring that ad hoc DEI indicators are also established for the monitoring and evaluation of the interventions. These indicators should prioritize gender equity and the meaningful engagement of LMIC stakeholders free from paternalistic and colonial attitudes [34].

### We are disconnected

OH should aim to establish a socioecological equilibrium that seeks a harmonious balance between human–animal–plant–environment interactions while recognizing the intrinsic value of all living things. This approach emphasizes the critical role of biodiversity, access to natural spaces and resources, and the interconnectedness of ecosystem health. OH will deliver its promise if it recognizes that the health of the environment is the critical foundation for the health and well-being of humans, animals and plants and that biodiversity is the very basis of ecosystem functioning and resilience while supporting health security [35, 36]. We are witnessing an ever-increasing disconnect between humans and nature in Western societies on the basis of an anthropocentric perspective and a narrow focus on the animal‒human interface to protect human interests [37]. Ecosystem interventions are designed and implemented primarily for human benefit without concern for how these interventions impact other species [38, 39]. Compounding this issue is a lack of awareness among the general population and even OH professionals of the importance of biodiversity for the prevention of health threats. This, combined with frequently difficult collaboration between the human, animal, and environmental health sectors; competing policy priorities; and limited financial resources, particularly in LMICs where biodiversity hotspots are concentrated, hinder biodiversity conservation and its integration into public policies and debates (40–42). For example, Madagascar, a biodiversity hotspot, has committed to the Paris Agreement through its second nationally determined contribution (NDC) to climate change, estimating the cost of related activities at $24.4 million USD. Owing to economic constraints, operationalization depends heavily on external financial support, with the government of Madagascar expected to contribute up to 3–4% of the indicated costs by 2030 through internal resources [43]. These examples highlight the pressing need for increased global investment and prioritization of biodiversity conservation within OH initiatives.

#### Panel 3: Avenues to support human–animal–environment equilibrium in One Health

Awareness of the disconnect between human beings and the ecosystems they live in and the understanding that ecoservices are essential for mutual survival constitute the first step toward meaningful change. Therefore, compelling actions to increase awareness among the general public, professionals and decision-makers about the importance of biodiversity and ecosystem health for human health security need to be effectively promoted at the global level, first by incorporating biodiversity conservation and ecosystem health concepts into educational and professional curricula and by supporting networks, research and programs that enhance biodiversity and ecosystems conservation to support human and animal health. For example, Biodiversa + is a co-funded biodiversity partnership, in the framework of the EU Biodiversity Strategy 2030, supporting research on biodiversity with a direct impact on research, policy and society [44]. At the policy level, emphasizing the coherence and coordination of biodiversity conservation and ecosystem health policies and initiatives with those related to human and animal health is fundamental [45].

### All life is of equal concern

For OH to be effective, it must promote stewardship and foster human responsibility to change behaviors and adopt sustainable solutions that prioritize animal welfare, plant health, and ecosystem integrity. This approach is essential for ensuring the well-being of current and future generations. Currently, human activity, mostly in industrialized countries, consumes resources equivalent to 1.6 earths, exceeding the capacity of ecosystems to regenerate [46]. A key implication of the OH approach is the urgent need to reduce human pressure on ecosystems and embrace a sustainable way of life. For example, providing a growing global population with healthy and safe diets from sustainable food systems is an urgent unmet need and requires a fundamental change of uncaring relationships with animals [47] and water and soil management. Intensive animal farming practices, characterized by high-density stocking of single-age, genetically homogeneous animals and poor biosecurity and diet, create conditions for pathogen spillover. Approximately 77% of livestock pathogens can infect multiple host species, including wildlife and humans, creating conditions for pathogen spillover [46]. Therefore, transitioning to healthy livestock production systems is essential to reduce these risks [49]. Within the OH framework, the interconnectedness of human and animal health has significant ethical implications, emphasizing equal concern for all life [50, 52]. Efforts to operationalize OH effectively and equitably face multiple barriers. This includes limited awareness of the link between animal welfare, human welfare and health security at both the institutional and community level, and economic incentives that perpetuate unsafe and unethical intensive farming practices to meet Western dietary requirements. At the community level, cultural beliefs, social norms, and individual behaviors such as traditional healing practices may hamper the acceptance and uptake of innovations, undermining ethical animal practices [53]. For example, in some parts of Uganda, traditional medicine involves the use of specific animal parts believed to possess healing properties, leading to mistreatment or even inhumane killing of animals, exacerbating animal welfare issues and human health risks [54]. These practices persist due to limited access to or trust in human medical facilities and failure to integrate traditional medicine into healthcare systems [55].

Another critical challenge is the poor awareness and engagement of younger generations in OH. Youth engagement is critical, as future leaders are responsible for addressing the complex threats they inherit. Sustainable implementation of equitable OH practices is impossible without actively involving young people in shaping and driving the OH approach [56].

#### Panel 4: Avenues to support stewardship and the responsibility of humans

To address these barriers, actions can be taken at the community and institutional level to support the adoption of sustainable and ethical OH solutions. In the community, targeted education campaigns can be implemented to promote awareness of the links among animal welfare, sustainability and human health security. Additionally, gender-related issues should be embedded in OH actions. In many East African pastoralist communities, women are responsible for the rearing of small livestock, such as poultry and goats; however, they often lack the authority to seek veterinary care and access training opportunities without the approval of male household members [57]. The OH for Humans, Environment, Animals and Livelihoods (HEAL) program, which was implemented in Kenya, Ethiopia and Somalia, has set up sustainable and demand-driven OH units that provide holistic and integrated health service delivery to the community focused on human and livestock health, natural resource management and gender equity [58]. HEAL built on the capacities of vulnerable pastoralist communities, services providers and regional institutions to ensure needs-based services provision with a bottom-up approach. In some areas, the presence of OH units is associated with improved health outcomes for both humans and livestock [59].

At the government level, there is a need to invest in the awareness of professionals working in relevant sectors about sustainable ethical solutions and to facilitate knowledge sharing and financial resource mobilization to align with national and global policies and targets [60].

To increase the rate of youth engagement efficiently, a two-pronged approach is recommended by establishing working relationships with today’s youth that enables them to be part of the identification of complex problems amenable to OH solutions and by working with education systems to ensure that divergent thinking (a thought process used to generate creative ideas by exploring many possible solutions) is encouraged in children rather than a focus on convergent thinking, which has contributed to disciplinary and sectoral siloes [61]. This approach can empower younger generations to play an active role in shaping sustainable OH practices.

### Holistic traditional knowledge systems are disrupted

OH promotes transdisciplinary and multisectoral collaboration, which includes both modern and traditional forms of knowledge and a broad representative array of perspectives. The notion that the wellbeing of an individual is directly connected to the wellbeing of the land has a long history in Indigenous knowledge systems [47, 62]. Indigenous knowledge systems are continuously disrupted by the effects of neo/colonization, which does not recognize the validity of local knowledge and instead supports the medicalization of health and positivist thinking. Indigenous people, in addition to holding secular knowledge that supports ecosystem health and sustainability, also hold 80% of the planet’s remaining biodiversity [63]. Many Indigenous communities face disproportionate health risks due to multiple factors, including contact with animals, environmental exposure, underrepresentation in decision-making processes, and limited access to healthcare. The concepts behind the OH approach are closely aligned with Indigenous ideologies that recognize the intrinsic relationships among the health of animals, people and the shared environment, and going forward, the refinement of OH concept definitions, guidelines and principles would benefit from active, respectful engagement with Indigenous peoples. [64], and OH concept definitions, guidelines and principles have commonly been developed with limited engagement of Indigenous peoples. Moreover, Indigenous representation within the evidence base and policy-making processes is commonly lacking [65]. Addressing this gap is critical for creating an inclusive OH framework that recognizes and respects Indigenous knowledge and contributes to health and sustainability.

#### Panel 5: Avenues to support the integration of holistic knowledge systems in One Health

The engagement of Indigenous perspectives in OH policies, programs and research should ensure the engagement of Indigenous people and their knowledge systems to improve the operationalization of the OH approach and enable it to draw on thousands of years of knowledge and experience. While examples are limited, evidence has shown the ability to address significant knowledge gaps through the adoption of Indigenous-led transdisciplinary approaches, for example, through an OH pilot study undertaken with the Aboriginal and Torres Strait Islander communities in Australia [66]. By adopting Indigenous research methodologies in OH, Indigenous voices and priorities were integrated throughout the research and subsequent recommendations, strengthening the OH evidence base [67]. Despite progress in the research field, there remains an urgent need to facilitate Indigenous engagement and leadership in international OH initiatives. Integrating First Nations perspectives into global OH policies and programs is essential to ensure that solutions are inclusive, equitable, and effective. Moreover, there is a rights-based need to decolonize conservation practices and recognize Indigenous people’s fundamental rights to protect and manage their own resources sustainably and ensure that local perspectives, knowledge and priorities lead conservation efforts [52].

### Pathways to support One Health to adhere to its DEI principles and support health security

Barriers to the full adherence of OH to its key underlining principles were inductively derived from the findings that emerged from this research and were grouped into five categories: mindset, behaviors and OH awareness; OH concept; structural; power dynamics; and OH governance and implementation. Mindset relates to a mental attitude that affects behaviors and it is influenced by the level of OH awareness, that is the knowledge and understanding of OH. Conceptual refers to ideas and principles that shape the OH concept. Structural barriers concern the way in which the parts of a system are coming together in relation to OH. Power dynamics are the ways in which power, influence, and authority are distributed and exercised. OH governance and implementation affect the way a system is managed.

**Fig. 2:**
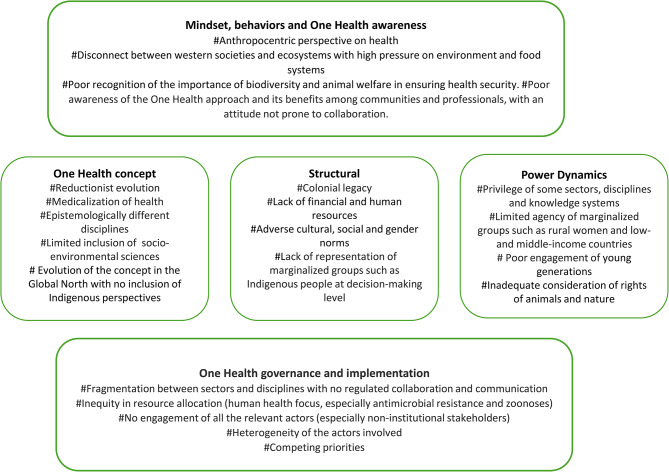
Categorization of the barriers to OH adherence to its key underling principles

Some underlying trends across these categories were identified, which highlighted the poor adherence of OH to its key underlying principles (Fig. 2). Overall, an anthropocentric perspective on health coupled with poor awareness of the OH approach and its benefits at both the individual and community level has supported the development of a flawed OH concept until the recent new definition [1]. This flawed approach is backed by structural barriers and inequitable power dynamics. As a result, OH governance and implementation is fragmented and does not effectively embrace all the needed sectors and disciplines, such as the environmental and social sciences; actors, from communities and young people to donors and OH professionals, including the private sector; and underrepresented groups, such as Indigenous peoples, farmers, fishers, representatives from low- and middle-income countries, and especially women across these groups.

These actors, groups, sectors, disciplines, and knowledge systems appear to be the most closely connected to the emergence of threats at the human–animal–environment interface [68–74] and are often excluded from OH initiatives, potentially impairing functional prevention and preparedness strategies. Such exclusion undermines the efficacy of prevention efforts that could save up to 37 billion USD globally by reducing the risk of pandemics and epidemics [75]. Therefore, supporting OH in adhering to its key underling principles while deploying preventive actions could significantly enhance health security.

Priority actions to address these barriers will require a change in mindset at all levels, from deep self-reflection on daily consumption choices to impactful global health decision-making. To drive this paradigm shift, targeted awareness and advocacy efforts are needed at both the institutional and noninstitutional levels through OH education and training for communities, students and professionals incorporating evidence-based benefits of engaging socioenvironmental sciences, communities, marginalized groups, biodiversity conservation, animal welfare, and Indigenous knowledge systems to develop an inclusive OH mindset.

At the conceptual level, aligning terminology and methodologies across different disciplines is critical for demonstrating how collaboration can lead to practical outcomes. Moreover, developing a more comprehensive and functional OH operational definition by engaging in its development the perspectives of all the relevant actors, groups, sectors, disciplines, and knowledge systems would also support OH operationalization.

At the structural level, engaging marginalized groups, such as rural women, Indigenous peoples, and LMIC representatives in OH decision-making processes at the local, national and global levels is crucial. This effort must be supported by sustained financial and human investments alongside targeted initiatives to increase representation.

Power dynamics would need to be leveled by allocating equitable resources to neglected disciplines and sectors, supporting inclusive policies, programs, and OH action informed by local priorities, including biodiversity conservation and animal welfare, alongside initiatives supporting agency building and knock down privileges.

These actions should support a unified OH governance and implementation embracing all actors, groups, sectors, disciplines, and knowledge systems, leveraging existing multisectoral, multidisciplinary and multigroup mechanisms and OH facilitators, and including specific DEI requirements for OH projects, programs, and policies. Further research is needed to demonstrate whether and how DEI strategies in OH work in comparable settings, what enabling conditions are necessary for success, what risks or trade-offs might emerge.

### Limitations

There are a few constraints to consider in interpreting our findings. Although the literature review was designed to be broad in scope, it is possible that some relevant sources were overlooked, particularly those not easily retrievable through standard search strategies. Additionally, the review provides limited insight into how DEI principles are currently being implemented in practice, reflecting a general lack of detailed documentation on operational aspects. Lastly, while the expert consultations included professionals from a range of backgrounds and regions, all participants were affiliated with the WfOH network. This may have introduced a degree of bias in perspective, despite the diversity represented within the group.

## Data Availability

The data are available from the corresponding author upon reasonable request and with the permission of coauthors and consulted experts.
